# Dissecting cell type–specific impact in lysosomal acid lipase deficiency–associated disorders

**DOI:** 10.1016/j.jlr.2023.100474

**Published:** 2023-11-14

**Authors:** Marit Westerterp, Fang Li, Hanrui Zhang

**Affiliations:** 1 Department of Pediatrics, University Medical Center Groningen, University of Groningen, Groningen, The Netherlands; 2 Cardiometabolic Genomics Program, Division of Cardiology, Department of Medicine, Columbia University Irving Medical Center, New York, NY, USA

**Keywords:** lysosomal acid lipase, Wolman disease, cholesteryl ester storage disease, liver, hyperlipidemia, enzyme replacement therapy

Lysosomal acid lipase (LAL), encoded by the *LIPA* gene, is the only known acidic lipase that hydrolyzes cholesteryl ester (CE) and triglycerides (TGs) in lysosomes. These lipids originate from endocytosis of lipoproteins or lipophagy of cytoplasmic lipid droplets (1). LAL is critical for lysosomal lipid breakdown (1). Loss-of-function mutations in the *LIPA* gene result in Mendelian disorders, leading to CE and TG accumulation in various tissues. Mutations causing the complete absence or near-absence of LAL activity lead to infant-onset Wolman disease, with major clinical features including hepatosplenomegaly, malabsorption, and adrenal insufficiency, presenting a median life expectancy of approximately 4 months. Mutations encoding LAL with up to ∼10% residual enzyme activity cause cholesteryl ester storage disease (CESD) with childhood- or adult-onset and highly variable disease manifestations, predominantly characterized by hypercholesterolemia and hypertriglyceridemia, hepatic steatosis and fibrosis, which, over time, may progress to severe liver damage (1).

*Lal*-deficient (*Lal*^*−/−*^) mice have been developed as a tool to study the systemic effects of *Lal* deficiency on different organ systems (2). *Lal*^*−/−*^ mice survive into adulthood with a life expectancy of 1 year, resembling CESD or late-onset LAL deficiency in humans. The major manifestations of *Lal*^*−/−*^ mice include hypercholesterolemia, hypertriglyceridemia, and hepatosplenomegaly. *Lal*^*−/−*^ mice also develop progressive loss of adipose tissues, while in humans, mesenteric lipodystrophy was only sporadically documented in CESD patients (3). These data suggest that *Lal*^*−/−*^ mice overall serve as a useful model to study the role of LAL in liver dysfunction although they do not replicate every aspect of human LAL deficiency; and the effects on liver dysfunction may not be due to cell-autonomous effects of LAL in hepatocytes alone.

The Kratky Laboratory has done extensive research on the role of LAL in the regulation of lipid and energy metabolism. In an earlier study, Leopold *et al.* (4) found that high-fat/high-cholesterol diet feeding increased hepatic CE accumulation in mice with hepatocyte-specific *Lal* deficiency, as well as hepatic inflammation and levels of blood transaminases, indicative of liver damage. In this issue of the *Journal of Lipid Research*, Bradić *et al.* (5) show that these phenotypes were not recapitulated in mice on a standard laboratory chow diet although they observed a nonsignificant trend toward increased hepatic total cholesterol (TC) accumulation and increased macrophage marker expression in the liver; the latter being assessed by proteomics profiling. The observations in mice with hepatic *Lal* deficiency were independent of age. Both young (9–11 weeks) and mature (50–60 weeks) mice showed only marginal alterations in the liver proteome and a nonsignificant trend toward increased hepatic TC, with no difference in hepatic TG (5). In contrast, on a chow diet, whole-body *Lal* deficiency caused hepatic TC and TG accumulation, along with a pronounced proinflammatory and profibrotic liver proteomic signature (5). The modest phenotype of hepatocyte-specific *Lal*^*−/−*^ mice on a chow diet may be due to the presence of approximately 40% remaining acidic CE hydrolase activity, which was only 15% in the livers of *Lal*^*−/−*^ mice (5). The remaining activity observed in the liver of *Lal*^*−/−*^ mice is likely due to other lipases that have noticeable activity even when exposed to the non-optimal acidic pH in the LAL assay, as previously implied during the validation of global *Lal*^*−/−*^ mice wherein an absence of both *Lal* mRNA and protein was confirmed, but not a complete deficiency in enzymatic activity when employing ^14^C-labeled cholesteryl oleate as the substrate (2). Together, these studies suggest that when the lipid substrate provided for LAL via the diet is limited, residual LAL activity in mice with hepatocyte-specific *Lal* deficiency suffices to protect the liver from extensive TC accumulation. These observations imply that patients with CESD or Wolman disease may benefit from maintaining a low dietary lipid intake, as is the current recommendation for these patients (1).

Bradić *et al.* (5) speculate that the residual LAL activity in mice with hepatocyte-specific *Lal* deficiency may originate from Kupffer cells and recruited macrophages, as well as circulating LAL produced by other organs. Indeed, LAL can be secreted via the endoplasmic reticulum—secretory pathway and subsequently recognized and reabsorbed by cells through mannose-6-phosphate receptor–mediated endocytosis. Once internalized, the enzyme is transported to lysosomes, where it remains enzymatically active and contributes to the degradation of lipid substrates (6). This mechanism is the premise for enzyme replacement therapy (ERT). Recombinant human LAL (sebelipase alfa) as an ERT is currently the preferential treatment option for CESD and Wolman disease (7).

Challenges remain as to how we can better understand which cells constitute the major source of LAL, which organs are particularly dependent on LAL, and how to leverage this knowledge toward therapeutic applications. In *Lal*^*−/−*^ mice, hepatocyte- or macrophage-specific transgenic expression of LAL-ameliorated pathogenesis in multiple organs, implying that hepatocytes and macrophages secrete LAL, which is subsequently taken up by LAL-deficient cells (8, 9). In patients with LAL deficiency, treatment options include liver transplantation and hematopoietic stem cell (HSC) transplantation, which, however, have limited success in halting multiorgan lipid accumulation and disease progression (1). While liver transplantation is a viable treatment for end-stage liver disease, recurrence of LAL deficiency-associated pathology is observed in postmortem examinations, evident by the presence of vacuolated, foamy macrophages in the allograft liver (7). HSC transplantation has demonstrated clinical efficacy via mechanisms involving repopulation of donor-derived macrophages and transfer of enzymes from donor-derived HSCs (10). The frequent culprits behind failure of HSC transplantation are delays in donor cell differentiation and implantation into target tissues (10). These data in mice and humans imply that macrophages and hepatocytes constitute a major source of LAL activity and that the liver is highly dependent on it.

Are cells continuously dependent on elevated levels of LAL to improve lysosomal lipid degradation? A systematic literature review suggests a potential correlation of reduced LAL activity with nonalcoholic fatty liver disease (NAFLD) pathogenesis (11), prompting the inquiry of whether increasing hepatic LAL can be beneficial in NAFLD. Unexpectedly, hepatic overexpression of LAL leads to increased liver immune cell infiltration and inflammation in mice fed a cholesterol-rich Western-type diet (12), suggesting that enhancing LAL alone may not correct NAFLD and may have unfavorable consequences. In addition, common genetic variation in *LIPA* that is associated with increased risks of coronary artery disease (CAD) is also linked to higher *LIPA* mRNA and LAL enzyme activity in monocytes and macrophages (13, 14). Although premature atherosclerosis has been documented in some CESD patients, common genetic variants in *LIPA* associated with CAD are not associated with lipid traits, suggesting myeloid cell–specific and lipid metabolism-independent genetic contributions (14). Does increased LAL in monocytes and macrophages lead to increased atherosclerosis, and what are the consequences of increased LAL activity in monocytes/macrophages for other tissues such as the liver? Further mechanistic studies of LAL in CAD will shed light on the benefits and risks in therapeutically targeting LAL, particularly in the context of LAL ERT that is currently approved for use in patients with LAL deficiency.

In summary, Bradić *et al.* (5) present important data showing that hepatocyte-specific LAL deficiency in mice was not sufficient to markedly alter the proteomic signature and lipid phenotypes when mice were fed a standard laboratory chow diet. This study highlights the role of cell–cell communication via transferable LAL enzyme in maintaining liver function when LAL activity is abolished in hepatocytes (Fig. 1).

**Fig. 1:**
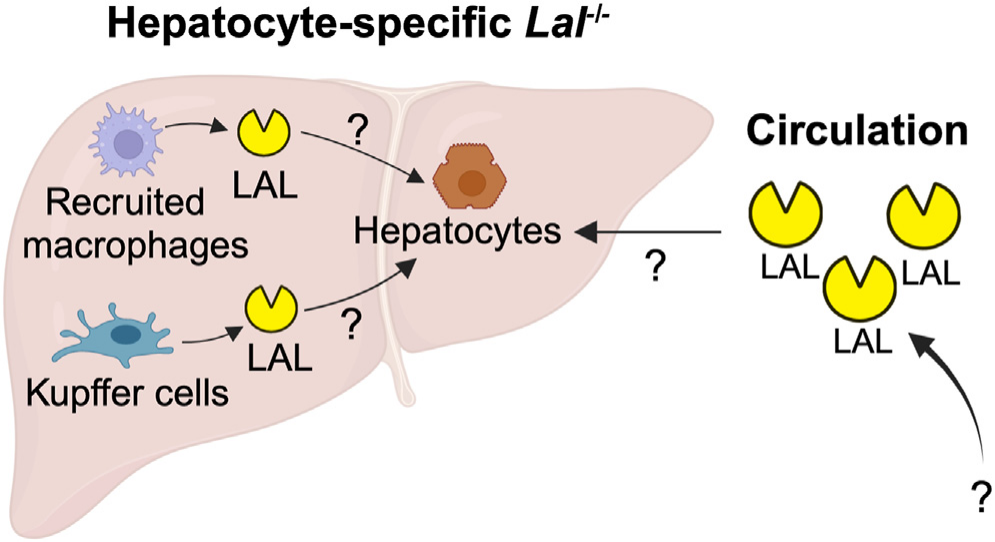
Cell type–specific impact of lysosomal acid lipase (LAL) deficiency and the potential role of cell–cell communication via transferable LAL enzymes. Bradić *et al.* found that hepatocyte-specific LAL deficiency in mice was not sufficient to markedly alter the hepatic proteomic signature and phenotypes when mice were fed a standard laboratory chow diet. The modest phenotype of hepatocyte-specific *Lal*^*−/−*^ mice on a chow diet is likely due to the presence of residual acidic CE hydrolase activity in liver lysates. The residual hepatic LAL activity may originate from Kupffer cells and recruited macrophages, as well as circulating LAL produced by other organs. The respective contributions of each source and the mechanism by which LAL enzymes are transferred to hepatocytes remain to be further elucidated. The figure is created with BioRender.com.

## Conflict of interest

The authors declare that they have no conflicts of interest with the contents of this article.
